# “When the happiness after childbirth turns into sadness…” Outpatient Therapeutic Program for the Treatment of Postpartum Mental Disorders

**DOI:** 10.1192/j.eurpsy.2026.11661

**Published:** 2026-07-31

**Authors:** V. Rößner-Ruff, J. Krieger, C. A. Penkov, K. Friedrich, M. Ziegenbein, A. Wilkening

**Affiliations:** 1 Research & Development; 2 Gender-sensitive day clinic for women, Wahrendorff Clinic, Sehnde, Germany

## Abstract

**Introduction:**

Pregnancy and the transition to parenthood pose significant physical and psychosocial challenges for women, increasing their vulnerability to mental health disorders. In addition to the common “baby blues,” which usually self-regulate, mental health disorders such as depression, anxiety, obsessive-compulsive disorder, PTSD, and psychosis can occur during the peripartum period. Persistent mental health problems in the mother affect not only her well-being but also that of her child, impacting the child’s emotional and behavioral development. Furthermore, postpartum mental health disorders often impact mother-child interaction. Despite the severity of the issue, shame and fear of stigmatization often cause it to be treated as taboo. Furthermore, treatment options are underrepresented.

**Objectives:**

The submitted contribution presents an outpatient therapeutic treatment program for women with postpartum mental health disorders and shares data on symptom severity collected using screening instruments.

**Methods:**

The Wahrendorff Clinic, a specialist psychiatric hospital in Lower Saxony (Germany) offers the outpatient therapeutic treatment program for women affected by postpartum mental disorders during and after pregnancy. The Depression Anxiety Stress Scale (DASS-P) and the Edinburgh Postnatal Depression Scale (EPDS) are used to evaluate symptom severity at the beginning of treatment.

**Results:**

The therapeutic treatment program focuses on providing therapeutic support and involves family members. It is administered by psychological psychotherapists, systemic therapists, and psychiatrists, who also provide medication. An evaluation is also carried out to assess the need for treatment, the severity of symptoms, and suicidal tendencies and risk factors. Other key aspects include providing referrals to inpatient parent-child facilities, breastfeeding support, early intervention services, parent support groups, and assistance in applying for domestic help or further outpatient psychotherapy. Preliminary results regarding mental health issues during the postpartum period, as measured by DASS-P and EDPS (n = 20), are also presented.

**Conclusions:**

The implications of the therapeutic treatment program are discussed in light of the preliminary results.

**Disclosure of Interest:**

V. Rößner-Ruff Employee of: No conflict value, J. Krieger Employee of: No conflict value, C. Penkov Employee of: No conflict value, K. Friedrich Employee of: No conflict value, M. Ziegenbein Employee of: No conflict value, A. Wilkening Employee of: No conflict value

